# Flawed research methods result in misleading conclusions

**DOI:** 10.1038/s41371-023-00818-w

**Published:** 2023-03-21

**Authors:** Rachael M. McLean, Feng J. He, Graham A. MacGregor

**Affiliations:** 1 Department of Preventive & Social Medicine, Dunedin School of Medicine, University of Otago, Dunedin, New Zealand; 2 Wolfson Institute of Population Health, Barts and The London School of Medicine and Dentistry, Queen Mary University of London, London, UK

**Keywords:** Risk factors, Hypertension

Delgardo-Ron et al. [1] use flawed methods to conclude that salt restriction in those with caloric deficiency may not be advisable. In a repeated cross-sectional analysis using publicly available National Health and Nutrition Examination Survey (NHANES) data 2007–2018, they restrict participants to those they believe are on calorie restricted diets and use regression methods to assess the association between sodium intake and hypertension.

Delgado-Ron et al. [1] use the energy balance method to identify people with calorie restricted diets- diets that are in energy deficit of at least 350 calories (equivalent to around 1465 kJ) per day. The use of energy balance methods are not new, and are designed to identify people likely to be under-reporting dietary intake, rather than those genuinely on an energy restricted diet [2, 3]. These methods used estimated energy expenditure from equations that estimate basal metabolic energy requirements from age, sex, weight and height plus self-reported energy expenditure (weekly vigorous and moderate physical activity) from the NHANES dataset. They then compare these with self-reported energy intake derived from 24 hour recall data.

Under-reporting in dietary surveys is common. Although the USDA multiple pass methods have been shown to reduce under-reporting compared with more traditional 24 h recall methods, under reporting is likely in around 25% of participants [4]. Under-reporting in dietary surveys is not random, and has been shown to be greater among older participants, those with lower education and those with a higher body mass index in NHANES data [4]. Under-reporting of sodium intake data has been shown to be much greater in overweight and obese subjects when validated with the gold standard 24 hour urine measure [5]. Further, those who under-report dietary intake are more likely to systematically under report foods perceived as unhealthy than healthy foods [6].

Participants included in this analysis (less than 10% of NHANES participants that completed dietary recalls between 2007 and 2018) are therefore likely to be a mixed group of those who under-reported energy intake, and those who genuinely have a calorie restricted diet- those who are overweight and attempting to lose weight, and those who are underweight [7], leading to bias. Both underweight and overweight participants are likely to have increased risk of co-morbidities and cardiovascular disease than those of normal weight [8].

Twenty four hour urinary excretion is the preferred method for assessment of dietary sodium intake in epidemiological studies. The Delgado-Ron et al’s statement that because they are examining blood pressure as the outcome, using dietary food recall survey data of sodium intake is more appropriate than assessing sodium excretion is false. Validation studies of food recall surveys use 24 hr urine sodium excretion as the standard of comparison [5, 9, 10]. Complete 24 hr urine samples on average represent 93% of ingested sodium at steady state but due to day-to-day fluctuations in diet and excretion multiple non-consecutive 24 hr urine samples are required to accurately assess usual sodium intake in individuals [11]. Food recall surveys have a variable but substantive systematic error underestimating sodium intake in the population (relative to 24 hr urine studies) and also have substantial random error in assessing individual sodium intake [5]. Based on a systematic review of the literature of studies comparing food recall surveys to 24 hr urine sodium excretion, an expert panel of a consortium of major international health and scientific organizations (TRUE consortium) specifically recommended food recall surveys to not be used to assess individual sodium intake [10]. The validity of using the NHANES food recall survey method of assessing sodium intake in individuals to associate with health outcomes has been reported as having a strong bias towards the null hypothesis based on the inaccuracies of the method (low correlation and attenuation factor) to assess sodium intake in individuals [5]. This is likely to at least partially explain the null findings of the Delgado-Ron analysis.

The choice of study design (repeated cross sectional analysis) can not be used for showing causal relationships in epidemiology. There is a high likelihood of residual confounding (given the marked differences between participants with hypertension and those without shown in Table 1 of Delgardo-Ron et al’s paper), and reverse causality (people with diagnosed hypertension may well be restricting both energy and sodium as a result of health advice based on current guidelines) [12]. Well conducted randomised controlled trials consistently show that reducing dietary sodium intake is associated with blood pressure lowering and reduced incidence of hypertension [13]. The totality of evidence strongly supports a reduction in salt intake across the whole population which will lower population blood pressure and reduce cardiovascular disease burden in the population.
